# Population structure and selective signature analysis of local sheep breeds in Xinjiang, China based on high-density SNP chip

**DOI:** 10.1038/s41598-024-76573-w

**Published:** 2024-11-15

**Authors:** Yanhao Li, Xiaopeng Li, Zhipeng Han, Ruizhi Yang, Wen Zhou, Yuwei Peng, Jianzhong He, Shudong Liu

**Affiliations:** 1 College of Animal Science and Technology, Tarim University, Alar, 843300 Xinjiang China; 2 Key Laboratory of Tarim Animal Husbandry Science and Technology, Xinjiang Production and Construction Corps, Alar, 843300 xinjiang China; 3 College of Life Science and Technology, Tarim University, Alar, 843300 Xinjiang China

**Keywords:** Sheep, SNP chip, Population genetic structure, Selective sweep, Computational biology and bioinformatics, Genetics

## Abstract

The frigid and droughty climate of Xinjiang in China has given rise to unique indigenous sheep breeds with robust adaptability and resistance. To investigate the genetic mechanism of adaptability of Xinjiang sheep to the local extreme environment, we conducted population genetic structure analyses for three native Xinjiang sheep breeds: Altay sheep (ALT), Bashbay Sheep (BSBC), and Duolang sheep (DLC), as well as two foreign sheep breeds: Suffolk and Dorset, using the Ovine Infinium HD SNP BeadChip(680 K). Our findings revealed distinct genetic and evolutionary histories between Xinjiang and foreign sheep breeds. Principal Component Analysis (PCA) and phylogenetic tree effectively differentiate these five sheep breeds based on their geographical origins, and the domestication level of Xinjiang sheep is comparatively lower than that of foreign sheep breeds. Furthermore, by utilizing three selective signature methods, namely Fixation Index (Fst), Cross Population Extended Haplotype Homozygosity Test (XP-EHH), and Nucleotide Diversity (π), we have successfully identified 22 potential candidate genes. Among these genes, there are *TBXT*, *PDGFD*, and *VEGFA*, which are closely related to tail type and lipid metabolism; *VIL1*, *SLC11A1*, and *ZBTB46*, which are associated with immune function; and candidate genes such as *BNC1*, *HDAC1*, and *BMP5*, which impact sheep reproductive traits. This study establishes a foundation for conserving and utilizing local sheep germplasm resources in Xinjiang and provides molecular insights into the genetic mechanisms governing sheep adaptation to extreme cold and arid environments.

**Supplementary Information:**

The online version contains supplementary material available at 10.1038/s41598-024-76573-w.

## Introduction

The domestication of sheep (Ovis aries) represents a significant milestone in human civilization, dating back to approximately 11,000 years ago in the Near East. It is widely accepted that modern domestic sheep descended from the Asian mouflon^1^. Sheep have undergone many different migrations and domestications throughout history, gradually differentiating into many contemporary sheep breeds with different genetic characteristics^2^^,^^3^. It is noted that these migration patterns are directly related to human activities^4^. Migration and domestication have both led to adaptive genetic changes, as evidenced by genetic diversity, gene flow, and selective pressures within sheep populations^5^^,^^6^, all of which play important roles in the development of current sheep breeds. China’s diverse climate and multifarious agricultural environments have shaped various excellent local sheep breeds. This is obvious not only in their unique physical characteristics, but also in their adaption to particular climates and agricultural conditions^7^.

Whole-genome variation detection to identify important genes related to economic traits in sheep is an important strategy for future genomics-assisted breeding. Based on the analysis of sequencing data from wild and domestic sheep, Cao et al^8^. demonstrated that introgression from wild sheep has enhanced the climate adaptability of domestic sheep, particularly the introgression in the specific region of *PADI2*on chromosome 2, which is associated with pneumonia resistance in domestic sheep. Li et al^9^. discovered numerous critical genes that affect sheep morphology and agricultural traits by in-depth sequencing and analysis of the genomes of several sheep breeds, including the *PDGFD* gene connected with fat deposition. In a detailed study of genomic variations in Tibetan sheep, which are widely distributed on the Qinghai-Tibet Plateau, researchers observed evidence of adaptive introgression from argali into Tibetan sheep, as well as strong selective signatures in genes related to hypoxia and UV signaling pathways (*HBB*, *MITF*, etc.) in Tibetan sheep^10^. Zhao et al^11^. collected samples from three native Chinese sheep breeds with extreme tail characteristics and performed a genome-wide scan, revealing multiple selected genomic regions, including genes related to tail morphology (*WDR92*, *TBX12*), reproduction (*PGRMC2*, *SPAG17*), and lipid metabolism (*JAZF1*). Based on information from a 50 K SNP chip in sheep, Zhang et al^12^. and Wang et al^13^. applied selective sweep analysis to screen candidate genes for desert adaptability (*SOD1*, *TSHR*, and *DNAJB5*) and reproductive traits (*SMAD2*, *ESR2*, and *HAS2*) in Xinjiang sheep. Additionally, Zhang et al^14^. categorized Xinjiang native sheep into six groups depending on their production performance and determined through environmental association analysis that the average temperature during the driest season and the precipitation season are the principal environmental factors influencing the adaptability of Xinjiang sheep.

The geographical environment in Xinjiang, China, exhibits significant variations, and the extreme climatic conditions and scarce natural resources pose constraints on the development of local agriculture and animal husbandry. However, under the long-term influence of both artificial and natural selection, native sheep in Xinjiang have gradually evolved survival tactics that are compatible with their environment. Nonetheless, there is a scarcity of genomic studies focusing on the environmental adaptability of sheep in Xinjiang. To delve into the molecular mechanisms behind native Xinjiang sheep’s resilience to severe settings, this study applied the Infinium HD SNP BeadChip (680 K) to conduct genotyping and genetic evolution analysis on five sheep breeds. Using the P-distance matrix, we created a neighbor-joining tree. Finally, the five sheep breeds were separated into two groups, and the selection sweep approach was used to screen for candidate genes connected with the environmental adaptation of the Xinjiang sheep population.

## Materials and methods

### Ethics statement

This study was accomplished in strict compliance with the Guidelines for the Biological Studies Animal Care and Use Committee, People’s Republic of China, and has received approval from the Animal Ethics Committee of the College of Animal Science and Technology, Tarim University (SYXK 2020-009). This study adhered to the relevant requirements of the ARRIVE Guidelines (https://arriveguidelines.org) in its design and execution.

### Animal samples collection

In this investigation, 307 sheep samples were collected, comprising 117 indigenous sheep from Xinjiang and 190 imported sheep from outside. 45 Bashbay sheep (BSBC) and 31 Duolang sheep (DLC) samples were obtained in the Tacheng and Kashgar regions of Xinjiang, respectively. 41 Altay sheep (ALT) samples were obtained from the Altay area of Xinjiang. On the other hand, 123 Dorset and 67 Suffolk samples were sourced from the Common Database. Genotyping and quality control

### Genotyping and quality control

The Phenol-Chloroform Protocol was utilized to extract DNA after collecting blood from sheep’s jugular veins. The extracted DNA samples are then amplified over the whole genome. The Ovine Infinium HD SNP BeadChip is used for genotyping purposes. Finally, all of the acquired SNP data were quality checked using the plink v1.90 software^15^. The quality control standards were as follows: (1) sample detection rate > 0.95, (2) SNP detection rate > 0.95, (3) minor allele frequency (MAF) > 0.05, and (4) Hardy-Weinberg equilibrium (HWE) *P*-value > 1 × 10^−6^.

### Analysis of population genetic structure

Following quality control, Principal Component Analysis (PCA) was completed with the plink v1.90 program, and the results were visualized using the ggplot2 package in R. VCF2Dis v1.50 (https://github.com/BGI-shenzhen/VCF2Dis) was utilized to compute the P-distance matrix, which serves as the foundation for ATGC: FastME (http://www.atgc-montpellier.fr/fastme) to construct a Neighbor Joining Tree (NJ tree). Subsequently, the web-based application iTOL^16^was used to improve and polish the aesthetic of the tree. We analyzed population structure using the Admixture software^17^, with K values ranging from 2 to 9. For this experiment, the optimal K value was chosen based on the lowest Cross Validation (CV) value.

### LD decay analysis

The LD coefficient (r2) was calculated for each pair of SNPs, with a maximum distance of 5000 kb between them. PopLDdecay v3.42^18^ was used to generate LD decay graphs according to the distance between SNPs.

### Selection sweep methods

To screen candidate genes associated with the adaptability of native sheep in Xinjiang to harsh environments, we categorized five sheep breeds into two groups: Group 1 encompassed three native sheep breeds from Xinjiang (ALT, BSBC, and DLC), whereas Group 2 encompassed two sheep breeds imported from abroad (Suffolk and Dorset). We then made use of three methods, fixation index (Fst), cross‑population extended haplotype homozygosity (XP-EHH), and nucleotide diversity (PI), to detect selective signatures between and within populations. We annotated the loci or windows within the 1% and 5% thresholds with reference to the sheep genome Ovis Oar_v4.0. Ultimately, a common set was obtained by intersecting the genes annotated.

Fst is an index used to measure the degree of genetic differentiation between populations. The calculation formula is as follows:

$$\:{F}_{st}=\frac{MSP-MSG}{MSP+({n}_{c}-1)MSG}$$

In this formula, MSP and MSG represent the mean square error within and between populations, respectively, while nc is the corrected average sample size of the entire population. We employed the VCFtools v0.1.16 software^19^ to compute the Fst between two populations, configuring the window size at 100Kb (--window-size 100000) and the step size at 25Kb (--window-step 25000). Subsequently, we leveraged the CMplot package in R to generate a Manhattan plot for visualization.

The XP-EHH analysis is a technique that relies on haplotype homozygosity to detect signatures of selection. In this investigation, XP-EHH analysis was performed on two sheep populations applying selscan v2.0.0^20^, and visualization was achieved with the CMplot package in R.

$$\:XPEHH=\text{l}\text{n}\left(\frac{{I}_{A}}{{I}_{B}}\right)$$

Here, I_A_ is the integration of the EHH statistic in the observed population with respect to genetic distance, while I_B_ signifies the integration of the EHH statistic in the reference population with respect to genetic distance.

Using the vcftools v0.1.16 software, we separately calculated the PI values for three breeds: DLC, BSBC, and ALT. The window size was set to 100Kb (-window-size 100000) and the step size was set to 25Kb (-window-step 25000). After sorting the results in ascending order, the top 1% and 5% scoring windows are identified as candidate regions.

$$\begin{aligned}PI=\sum_{j=i}^{S}{h}_{j}\end{aligned}$$

In this equation, S and h_j_ represent the number of segregating sites and the j segregating site’s heterozygosity, respectively.

**Fig. 1:**
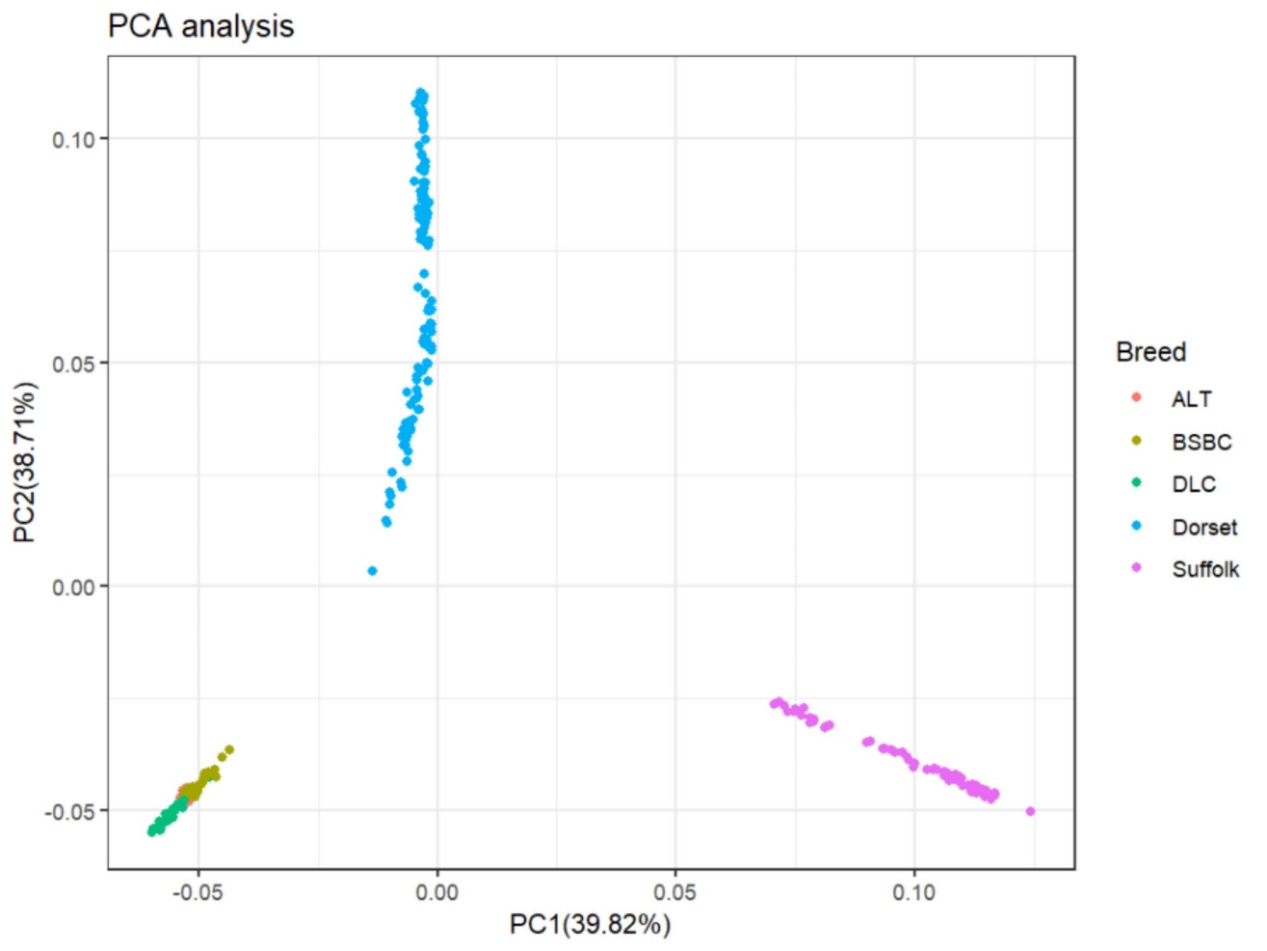
PCA of five sheep breeds, with the x and y axes representing principal components 1 and 2, respectively.

### Enrichment analysis of candidate genes

Gene Ontology (GO) and Kyoto Encyclopedia of Genes and Genomes (KEGG) analyses^21^were performed on genes within a 5% threshold via the online tool DAVID^22^. The KEGG pathway database originates from Kanehisa Laboratories (www.kegg.jp/kegg/kegg1.html). Terms and pathways with a *P*-value < 0.05 were considered significantly enriched. The enrichment results are visualized using ggplot2 in R.

**Fig. 2:**
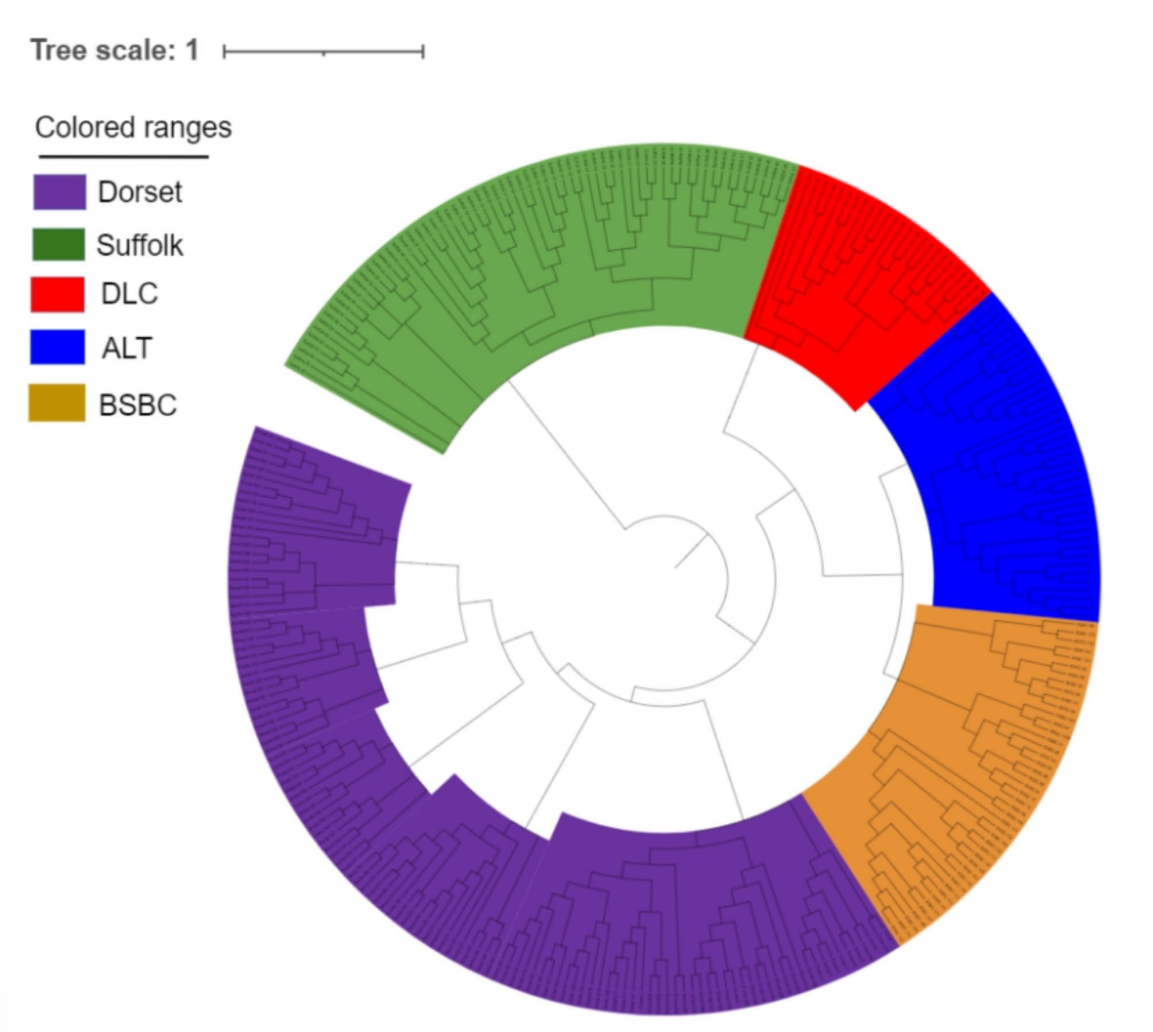
NJ tree of five sheep breeds, with green representing Suffolk, red representing DLC, blue representing ALT, purple representing Dorset, and yellow representing BSBC.

**Fig. 3:**
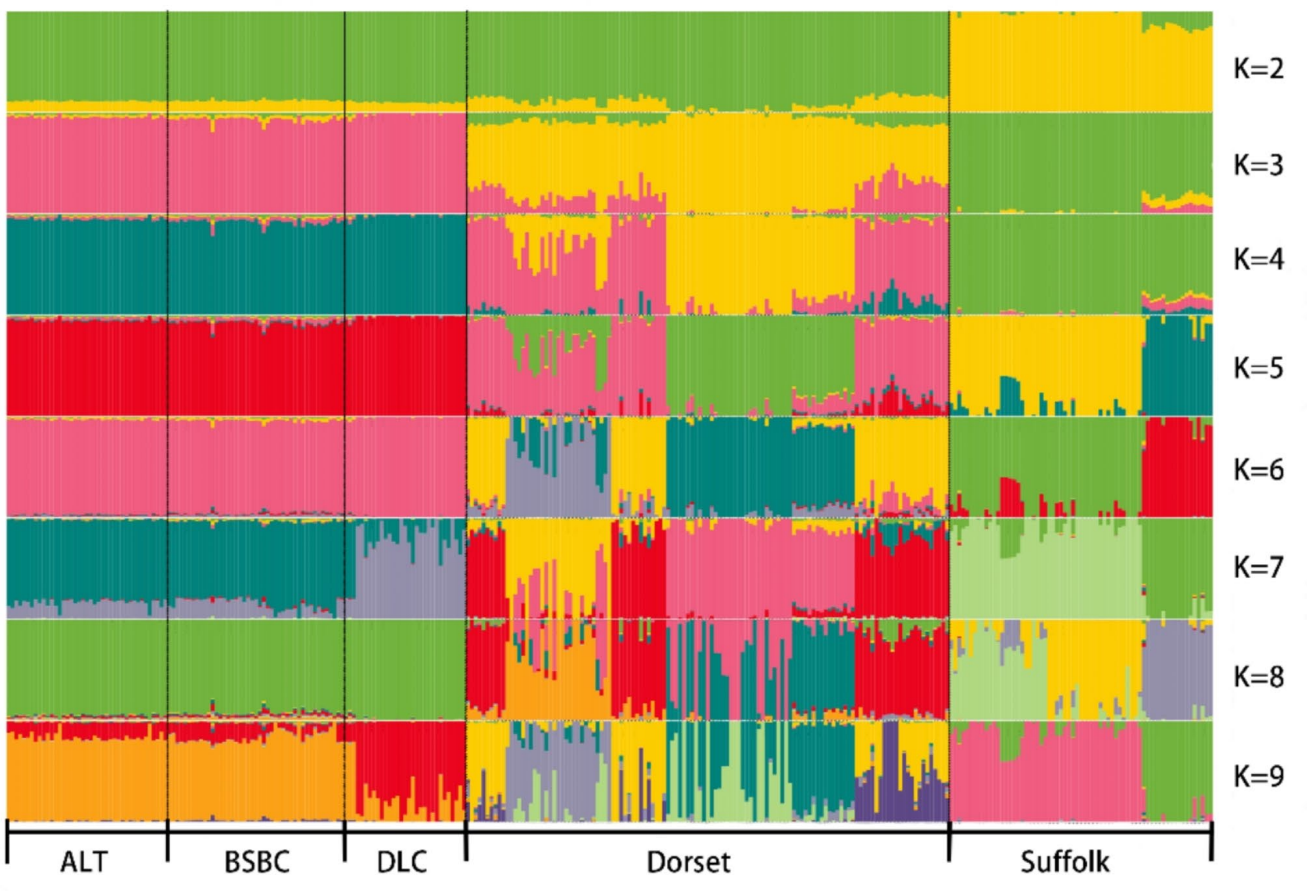
Admixture diagram of five sheep breeds, K = 2–9, with different colors representing different ancestral components.

## Results

### Sample quality control

There were 524,795 SNP loci and 307 samples in total in this investigation. 307 samples and 479,298 SNP loci were kept for further analysis following quality control. Of these, chromosome 1 had the greatest number of loci (54,640), followed by chromosome 2 (48,343). Chromosome 24 had the fewest loci, totaling 8,531.

**Fig. 4:**
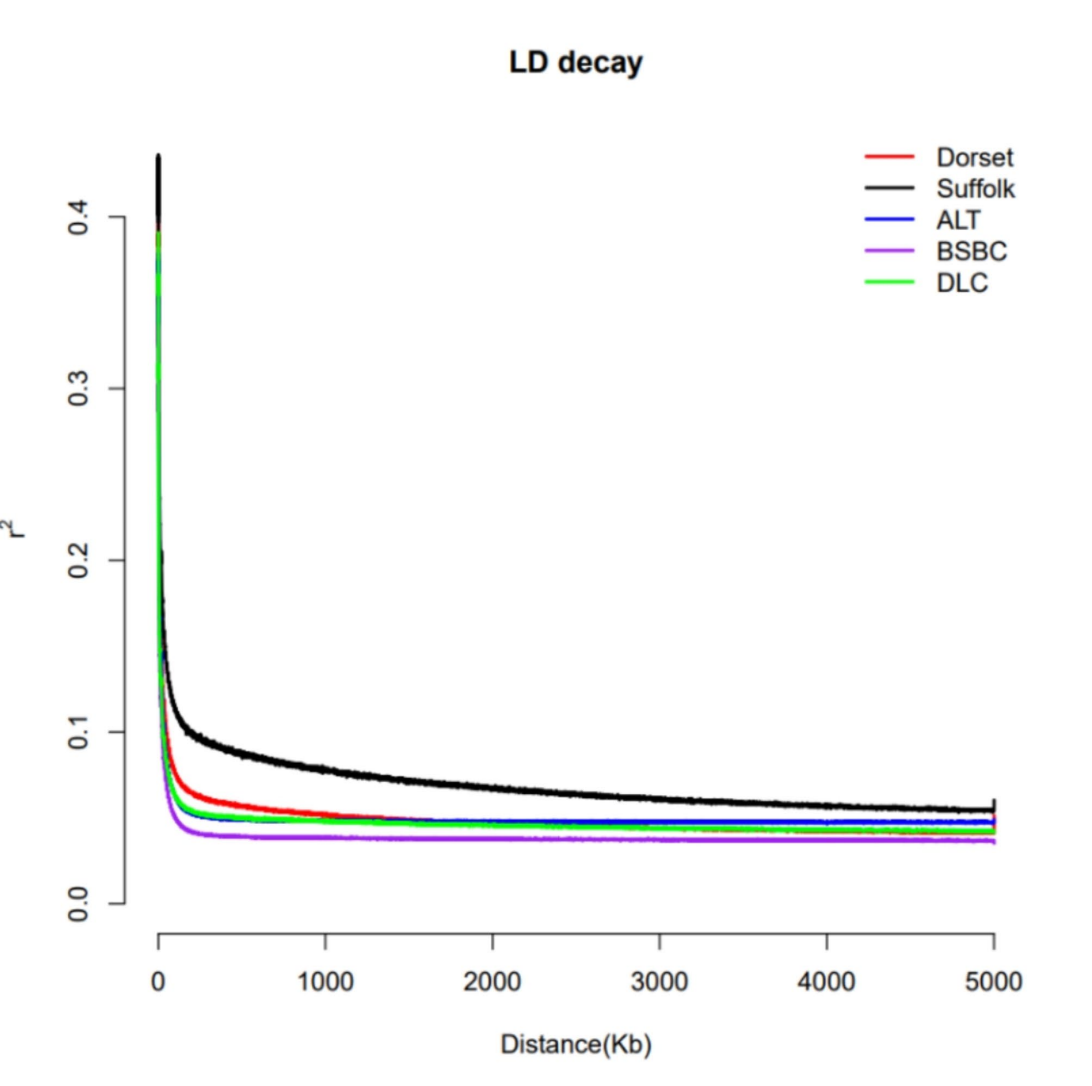
LD decay plots for five sheep breeds, with the X-axis representing the physical distance between markers and the Y-axis representing r^2^.

### Population genetic structure

The PCA was performed on five sheep breeds (Fig. 1). The outcomes illustrated significant variations in the principal components between the local sheep population in Xinjiang and the two foreign sheep populations. Notably, the Xinjiang sheep breeds exhibit a higher degree of similarity among themselves, clustering closely together. The NJ tree, as shown in Fig. 2, separates the five sheep breeds based on their geographical locations, with Dorset, Suffolk, and Xinjiang sheep each occupying distinct nodes, consistent with the PCA results. Compared to DLC, which inhabits the desert environment of southern Xinjiang, ALT and BSBC, located in northern Xinjiang, exhibit a closer genetic distance. In the admixture analysis depicted in Fig. 3, we ascertained that the experimental CV value reached its minimum when K = 7, indicating a distinct differentiation between Xinjiang sheep and foreign breeds. When K ranged from 2 to 6 and 8, Xinjiang sheep exhibited nearly identical ancestral components, standing apart from foreign sheep varieties. However, when K was set to 7 and 9, DLC from southern Xinjiang and ALT, BSBC from northern Xinjiang exhibited certain differences, which align with the findings of the NJ tree.

### LD decay analysis

The results of LD decay analysis are presented in Fig. 4. Within the range of 0-1000 kb, as the distance between markers continues to increase, the r^2^ value of all sheep breeds exhibits a decreasing trend. However, the decreasing trend in Suffolk is significantly slower than that of other sheep breeds, followed by Dorset. BSBC displays the fastest decreasing trend.

**Fig. 5:**
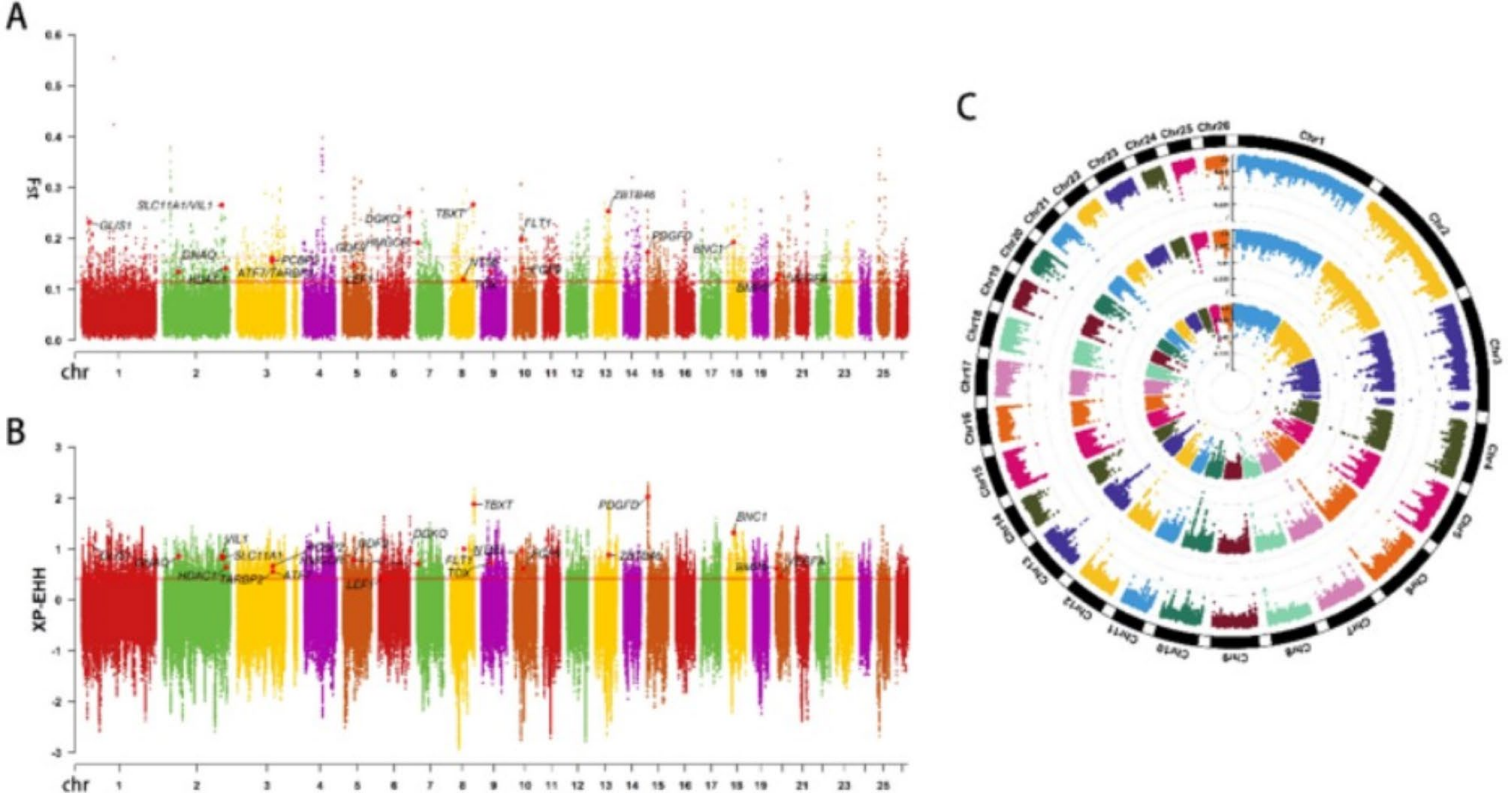
**A** and **B** are the resuts of Fst and XP-EHH analyses, respectively, with the red lines representing the threshold of 1% and 5%. **C** is the results of PI analysis for three Xinjiang sheep populations, which are DLC, ALT and BSBC from inside to outside.

### Selective signature

**Fig. 6:**
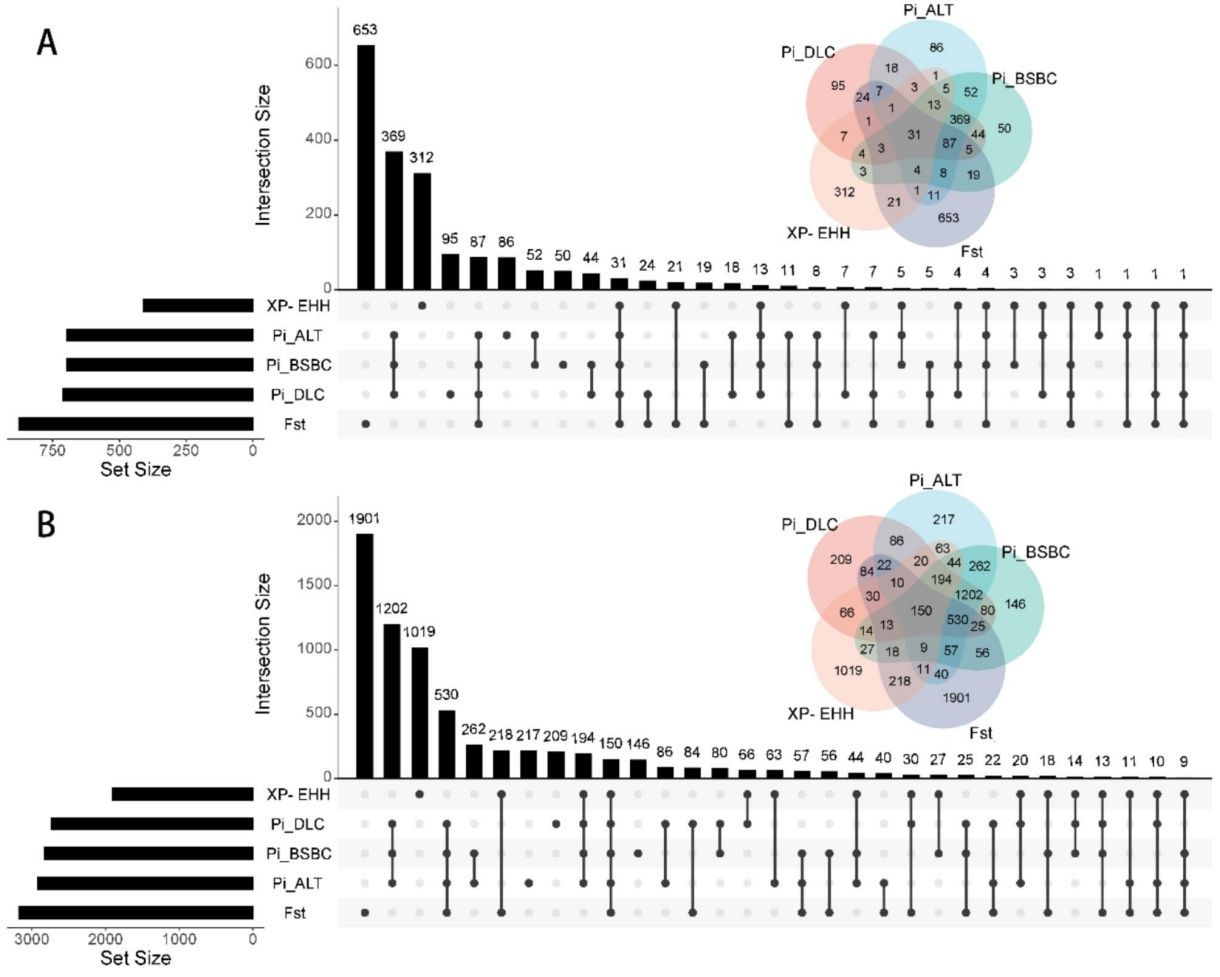
**A** and **B** represent the intersection of genes under the thresholds of 1% and 5%, respectively.

After the selection sweeps, the Manhattan plots were shown in Fig. 5. The three methods revealed 31 common genes in the top 1% region (Fig. 6A, Supplementary Table S1-S6) and 150 common genes in the top 5% region (Fig. 6B, Supplementary Table S7-S12). These include 22 candidate genes associated with tail morphology and energy metabolism (*TBXT*, *GLIS1*, *DGKQ*, *PDGFD*, *VEGFA*, *FLT1*, *FGF9*, *HMGCR*, and *ATF7*), immunity (*VIL1*, *SLC11A1*, *ZBTB46*, *LEF1*, *TOX*, *PCBP2*, *TARBP2*, and *NT5E*), and reproduction (*BNC1*, *HDAC1*, *BMP5*, *GDF9*, and *GNAQ*) in Xinjiang sheep.

### Enrichment analysis of candidate genes

**Fig. 7:**
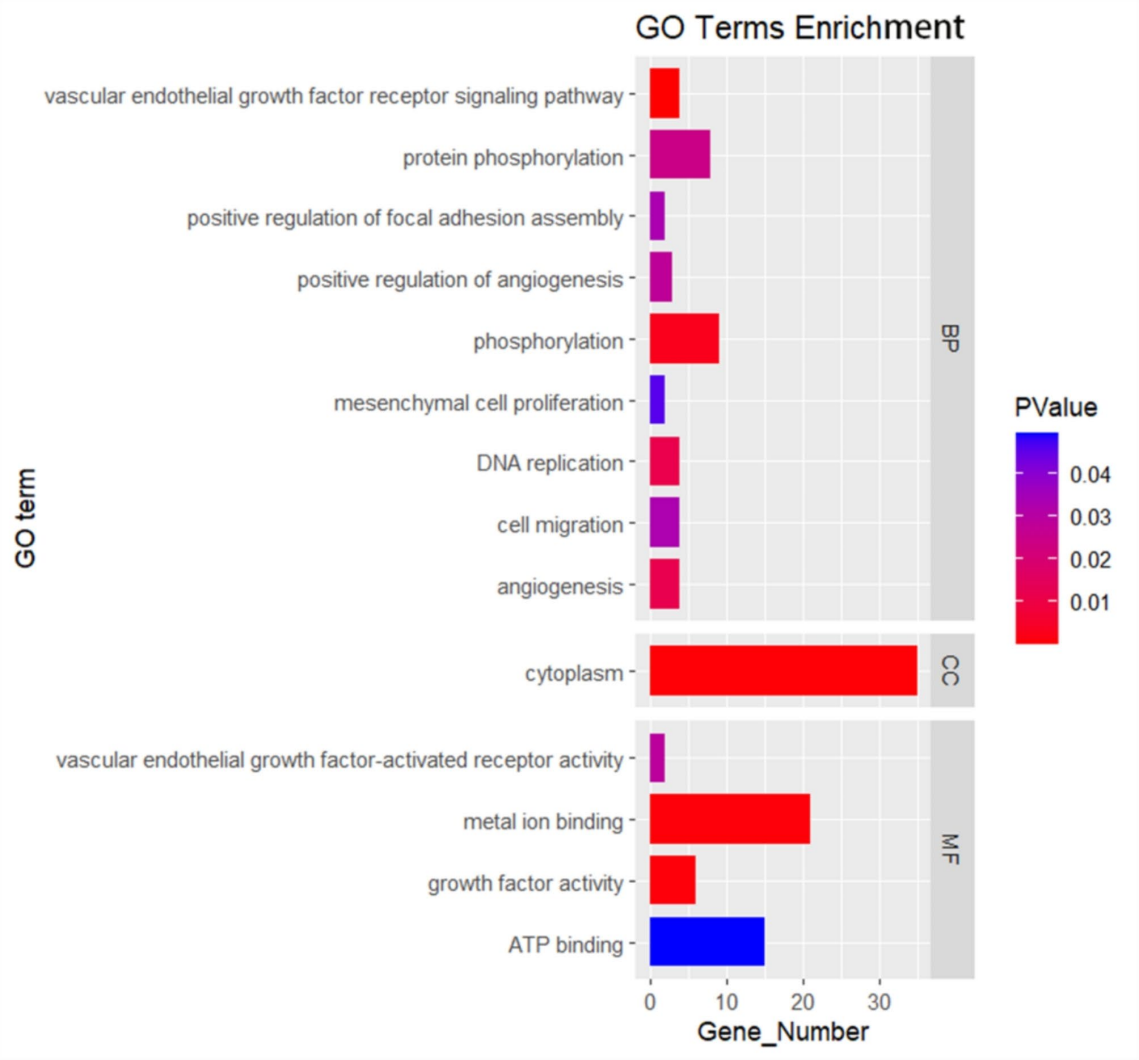
Visualization of GO enrichment analysis of 150 genes, *P* < 0.05.

**Fig. 8:**
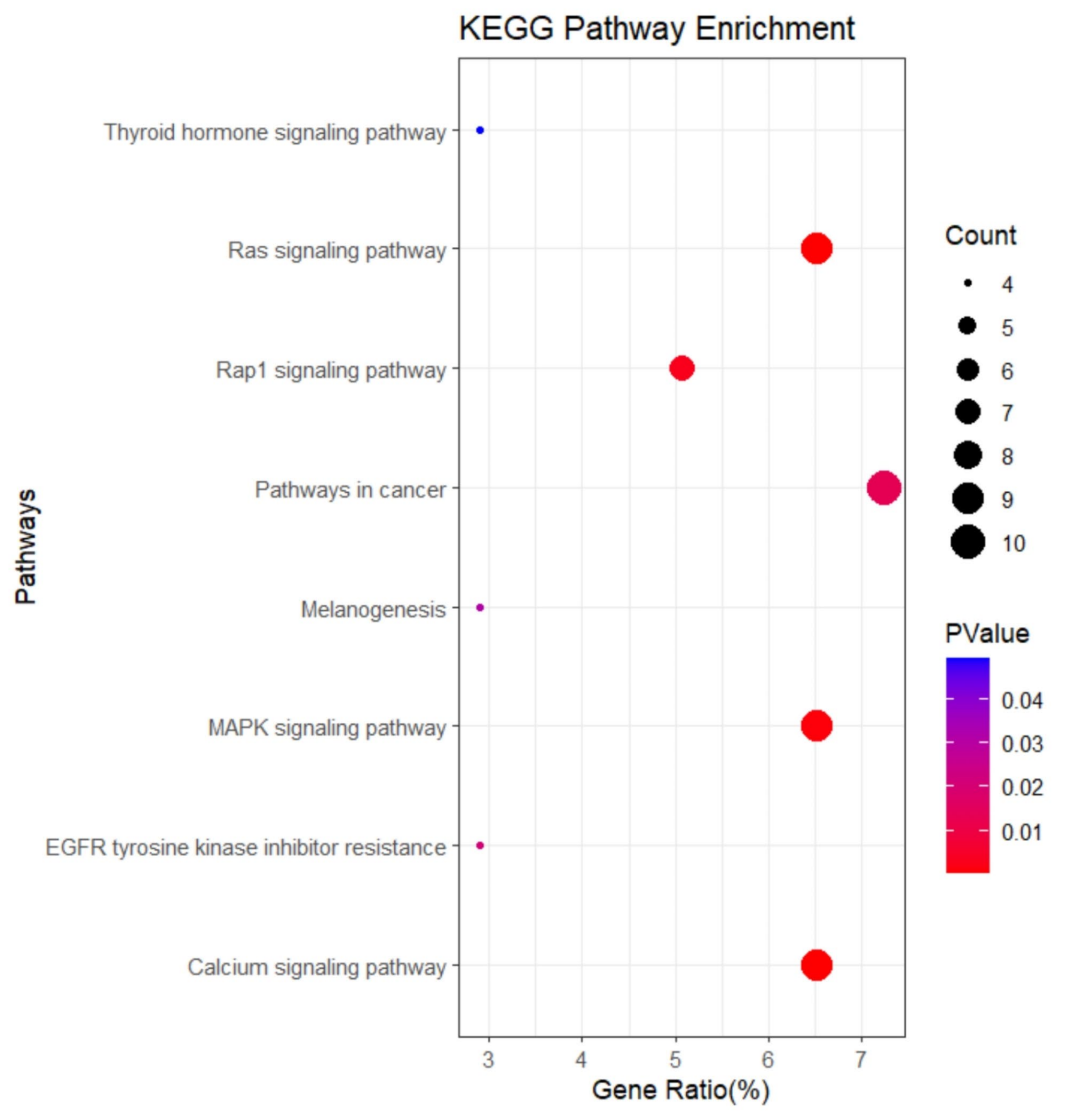
Visualization of KEGG enrichment analysis of 150 genes, *P* < 0.05.

We executed GO and KEGG enrichment analyses on the 150 genes in the intersection. In the GO enrichment analysis, a total of 14 significantly enriched terms (*P* < 0.05) were uncovered (Fig. 7, Supplementary Table S13). An intriguing phenomenon is that six of these terms are linked to angiogenesis and growth factors, encompassing genes such as *PGDFD*, *VEGFA*, *FLT1*, and *FGF9*, which are associated with adipogenesis in Xinjiang sheep. The KEGG analysis revealed 8 significantly enriched pathways (Fig. 8, Supplementary Table S14), including the Ras signaling pathway, Calcium signaling pathway, MAPK signaling pathway, and Rap1 signaling pathway, among others.

## Discussion

### Population genetic structure

Leveraging genome-wide SNP molecular markers, this study assessed the population genetic structure of five different sheep breeds. The five breeds were divided into three subgroups based on the PCA results. This is because Suffolk and Dorset originate from Britain and Australia, respectively, while DLC, BSBC, and ALT are native to Xinjiang. Notable differences in their genomes suggest that the agricultural climate of Xinjiang has fostered distinct sheep breeds that are distinguishable from those in other areas.

We established an NJ tree for the five breeds based on the P-distance matrix. The clustering of Xinjiang sheep breeds at a common node suggests a genetic proximity among them. However, compared to the DLC in the southern Xinjiang region, the NJ tree reveals a closer genetic distance between BSBC and ALT. Both ALT and BSBC, originating from northern Xinjiang, exhibit a closer kinship with Kazakh sheep^23^, Which indicates that the different ecosystems between the northern and southern regions of Xinjiang have also led to disparities among the sheep populations.

The admixture analysis demonstrates that Xinjiang sheep possess fundamentally distinct ancestral components from their foreign counterparts. The Dorset and Suffolk breeds manifest a more diverse ancestry when compared to Xinjiang sheep, suggesting that the unique geographical and climatic conditions in Xinjiang may have imposed certain limitations on sheep migration and admixture, thereby causing a relatively homogenous genetic background for the local sheep population. Consequently, there is a need for proactive efforts in introducing foreign genetic resources and fostering increased genetic exchange among different sheep populations. The discovery that BSBC and ALT share similar ancestral components and can be distinguished from DLC when K = 7 and 9 may stem from historical hybridization events. DLC, a breed developed through careful breeding over years from the crossbreeding of Afghan sheep with local sheep in Xinjiang^24^, which may have contributed to its divergence from other sheep breeds. Our findings align with the research conducted by Wei et al^25^. further corroborating the conclusions drawn from the evolutionary tree.

### LD decay analysis

The LD decay analysis unveils that the Suffolk breed exhibits the slowest LD decay rate, presumably attributed to its high domestication level and intense selection pressure as an preeminent breed of meat sheep. Conversely, the Xinjiang sheep population, particularly the BSBC sheep, displays a faster LD decay rate in contrast with foreign sheep breeds, implying a potentially lesser degree of genomic selection. Consequently, it is imperative to intensify the breeding efforts of Xinjiang sheep.

### Analysis of selective signature

Selection can occur as positive selection, wherein the frequency of favorable mutations increases, or as negative selection, wherein the frequency of deleterious mutations decreases. While the Fst analysis, based on allele frequencies, is a method to measure the degree of genetic differentiation between different populations, it is unable to determine the direction of selection^26^. Conversely, the XP-EHH analysis, by comparing the extended haplotype lengths surrounding specific alleles in diverse populations, can identify regions potentially under positive selection^27^. PI, also known as nucleotide diversity (π), typically signifies a reduction in genetic diversity within a region when its value is low, which often indicates the presence of selective pressures. Therefore, in selection scans, we have utilized these three approaches to complement each other. Windows scoring within 1% and 5% across all three methods are considered as potential areas under positive selective pressure in Xinjiang sheep. After conducting the enrichment analysis, genes associated with angiogenesis have been selected, particularly *PGDFD*, *VEGFA*, *FLT1*, and *FGF9.* We speculate that these genes may serve as crucial determinants in the regulation of fat deposition and local environmental adaptation in Xinjiang sheep.

### Genes associated with tail traits and lipid metabolism

Climatic-mediated selective pressures have profound impacts on the genetic variation and evolution of sheep^28^. Some animals adapt to extreme environments, such as low temperatures, hypoxia, and food scarcity, by adjusting their energy metabolism and increasing fat reserves. For instance, the fat-tailed trait of sheep is a case in point^29^. Under a threshold of 1%, we have recognized three genes, namely *TBXT*, *GLIS1*, and *DGKQ*, that are associated with tail type and fat metabolism in Xinjiang sheep. *TBXT* (T-box transcription factor T) plays a crucial role in the developmental process of mammals, especially in the development of the tail and spine. Researches have corroborated that the polymorphism of the *TBXT*gene is correlated with the short-tailed phenotype in sheep^30^^,^^31^. However, the genetic mechanism underlying the tail type variation in sheep caused by this gene still requires further investigation^32^. *GLIS1*, a pro-adipogenic factor, possesses the function to promote the formation of brown adipose tissue^33^. Given the unique role of brown adipose tissue in energy metabolism and thermoregulation, the *GLIS1*gene may influence fat deposition in the tail of sheep. Luo et al^34^. reached a consensus with our findings based on the selective sweep analysis of Mongolian sheep and Small Tail Han sheep. *DGKQ*(Diacylglycerol kinase theta) regulates the concentration of diacylglycerol (DAG) through the phosphoinositide cycle^35^. DAG, as a second messenger, can activate protein kinase C (PKC) and further affect downstream insulin signaling and glucose metabolism^36^. In GO enrichment analysis, *DGKQ* was found to be involved in phosphorylation and ATP binding pathways, indicating that this gene plays a significant role in adipose and energy metabolism in Xinjiang sheep.

Additionally, under the threshold of 5%, we have also identified genes such as *PDGFD*, *VEGFA*, *FLT1*, *FGF9*, *HMGCR*, and *ATF7* as having substantial influence on lipid metabolism in sheep. Among them, *PDGFD*, *VEGFA*, *FLT1*, and *FGF9*are all implicated in angiogenesis. Given the close interdependence between angiogenesis and adipogenesis^37^^,^^38^, these genes may regulate fat deposition in sheep through angiogenesis. *PDGFD*is a gene that has been extensively studied in sheep. Its expression in fat-tailed sheep is significantly higher than that in thin-tailed sheep, indicating a close correlation with adipose deposition in sheep’s tail^39^^,^^40^. *VEGFA*(Vascular Endothelial Growth Factor A) is necessary not just for angiogenesis, but also for the normal development and function of adipose tissue^41^. A meta-analysis has confirmed that *VEGFA*is associated with the deposition of adipose tissue in the human waist and hips^42^. Zhao et al^43^. propose that this gene could serve as a promising candidate for sheep tail type based on a selective scanning study. *FLT1* is the receptor for *VEGFA*. it possesses a crucial function in regulating angiogenesis^44^. Knockdown of *FLT1* has been demonstrated to enhance the browning of white adipose tissue in mice, likely attributed to, in part, the increase in adipose tissue vascularization mediated by *FLT1*^45^^,^^46^. *FGF9* is capable of inducing the expression of *UCP1*(Uncoupling Protein 1) in brown adipose tissue, playing a pivotal role in thermogenesis within this tissue and overall energy metabolism^47^. *HMGCR*, a classical rate-limiting enzyme in cholesterol biosynthesis, has emerged as a target for the treatment of hyperlipidemia^48^. The phosphorylation status of *HMGCR* regulates cholesterol production, thereby facilitating the modulation of lipid homeostasis in the organism. *ATF7* participates in various physiological processes of adipocytes by regulating the expression of *ISG* and *Ucp1*, as well as promoting the browning of white adipose tissue^49^. Liu et al^50^. proved that *ATF7* can enhance intramuscular fat production in sheep.

### Genes associated with immunity

We have further uncovered candidate genes associated with immune traits in sheep, which are potentially critical factors in enhancing the resilience of Xinjiang sheep to adverse conditions. These genes comprise *VIL1*, *SLC11A1*, and *ZBTB46*, located beneath the 1% threshold, as well as *LEF1*, *TOX*, *PCBP2*, *TARBP2*, and *NT5E*, among others, situated under the 5% threshold. The *VIL1*gene generates a protein that is intimately affiliated with intestinal epithelial cells. Nagaraj et al^51^. have uncovered the potential significance of *VIL1*in sheep’s defense against Haemonchus contortus infection through proteomic analysis, which implies a strong association with the animal’s resilience to gastrointestinal nematodes^52^. *SLC11A1* (Solute Carrier Family 11 Member 1) is involved in iron metabolism and the host’s resistance to certain infectious diseases. Research has revealed that *SLC11A1*can influence the susceptibility or resistance of goats to diseases such as brucellosis, pneumonia, and paratuberculosis^53^^,^^54^^,^^55^. *ZBTB46*has the capacity to regulate the inflammatory activity of ILC3^56^contributing significantly to the maintenance of intestinal health and stability^57^. *LEF1* and *TOX*, both genes, hold a pivotal position in regulating thymocyte selection, where *TOX* exerts a promotional effect on the expression of *LEF1*^58^. In the crucial stages of early T cell development and differentiation, these genes occupy a significant role. Specifically, *LEF1*, in collaboration with transcriptional factors such as TCF-1, facilitates the determination of the CD4 + T cell lineage and solidifies the identity of CD8 + T cells^59^. Moreover, *TOX*orchestrates the differentiation of CD4 + and CD8 + lineages throughout various stages of T lymphocyte development. Notably, the absence of TOX in mice (TOX-/-) leads to a pronounced reduction in the number of CD4 + T cells^58^. The roles of *PCBP2* and *TARBP2* are primarily manifested in their regulation of the antiviral immune response in the body. *PCBP2*inhibits the production of downstream IFN and other antiviral cytokines by negatively regulating the MAVS and cGAS-mediated signaling pathways^60^^,^^61^. In the case of Foot-and-Mouth Disease Virus (FMDV), the VP0 protein interacts with *PCBP2*to suppress the IFN-β pathway, thereby promoting FMDV replication^62^. *TARBP2*exhibits a similar role in antiviral activity, inhibiting the production of IFN-β through the MAVS pathway^63^. *NT5E*encodes a cell surface phosphatase, which functions to convert adenosine monophosphate (AMP) into adenosine. Adenosine, subsequently, exhibits its immunosuppressive effect by interacting with immune cells^64^.

### Genes related to reproduction

The genes primarily associated with reproductive traits in sheep include *BNC1*, *HDAC1*, *BMP5*, *GDF9*, and *GNAQ*. The discovery of these genes provides molecular evidence for the reproductive potential of Xinjiang sheep. In male reproductive physiology, *BNC1* directly binds to the promoter regions of crucial spermatogenesis genes such as *Ybx2* and *Papolb*, thereby promoting the transcription of these genes^65^. In females, the absence of the *BNC1*gene can lead to premature activation of oocytes and excessive follicular atresia^66^. *HDAC1*engages in epigenetic regulation through histone deacetylation, exerting a significant influence on promoting the development of ovine oocytes^67^. *BMP5* and *GDF9*, both belonging to the transforming growth factor-β (TGF-β) superfamily, play pivotal roles in the reproductive processes of mammals. *BMP5*expression in rat ovaries has been shown to stimulate granulosa cell proliferation and inhibit progesterone secretion^68^. Further studies have established a correlation between this gene and reproductive traits in goats^69^^,^^70^. *GDF9*is an indispensable factor in ovarian development and follicular maturation^71^. Certain mutations in this gene have been associated with an increase in ovulation rate and litter size in sheep^72^. The estrus cycle of animals is regulated by the endocrine system of the hypothalamus-pituitary-gonadal axis. *GNAQ*(G protein subunit alpha q) can directly or indirectly regulate the secretion of gonadotropin-releasing hormone (GnRH) in the hypothalamus of sheep through various pathways, thereby affecting the estrus cycle of sheep^73^.

## Conclusion

This study conducted a genetic evolution analysis of three native sheep breeds in Xinjiang and two foreign sheep breeds using a high-density sheep SNP chip. Additionally, we constructed a selection signature map for the three native sheep breeds in Xinjiang and analyzed candidate genes related to the local environmental adaptability of Xinjiang sheep (tail type, lipid metabolism, immunity, and reproduction) as well as their potential regulatory mechanisms. In future research, we will further delve into the specific genetic mechanisms of these genes, aiming to provide valuable references for subsequent sheep breeding and genetic improvement, and to provide solid theoretical support for enhancing the adaptability and productivity of sheep.

## Electronic supplementary material

Below is the link to the electronic supplementary material.

Supplementary Material 1

## Data Availability

If you require access to the relevant data used in this article, you may directly contact the corresponding author for acquisition.
